# Reduced middle ear infection with non-typeable *Haemophilus influenzae*, but not *Streptococcus pneumoniae*, after transition to 10-valent pneumococcal non-typeable *H. influenzae* protein D conjugate vaccine

**DOI:** 10.1186/s12887-015-0483-8

**Published:** 2015-10-19

**Authors:** Amanda Jane Leach, Christine Wigger, Kim Hare, Vanya Hampton, Jemima Beissbarth, Ross Andrews, Mark Chatfield, Heidi Smith-Vaughan, Peter Stanley Morris

**Affiliations:** Menzies School of Health Research, Charles Darwin University, Darwin, Northern Territory Australia; Royal Darwin Hospital, Darwin, Northern Territory Australia

**Keywords:** Nasopharynx, *Streptococcus pneumoniae*, Nontypeable *Haemophilus influenzae*, Otitis media, child, Indigenous, Pneumococcal vaccines, Prevalence, Surveillance, Risk factors

## Abstract

**Background:**

In October 2009, 7-valent pneumococcal conjugate vaccine (PCV7: Prevenar^TM^ Pfizer) was replaced in the Northern Territory childhood vaccination schedule by 10-valent pneumococcal *Haemophilus influenzae* protein D conjugate vaccine (PHiD-CV10; *Synflorix™* GlaxoSmithKline Vaccines). This analysis aims to determine whether the reduced prevalence of suppurative otitis media measured in the PHiD-CV10 era was associated with changes in nasopharyngeal (NP) carriage and middle ear discharge (ED) microbiology in vaccinated Indigenous children.

**Methods:**

Swabs of the NP and ED were collected in remote Indigenous communities between September 2008 and December 2012. Swabs were cultured using standardised methods for otitis media pathogens. Children less than 3 years of age and having received a primary course of 2 or more doses of one PCV formulation and not more than one dose of another PCV formulation were included in the primary analysis; children with non-mixed single formulation PCV schedules were also compared.

**Results:**

NP swabs were obtained from 421 of 444 (95 %) children in the PCV7 group and 443 of 451 (98 %) children in the PHiD-CV10 group. Non-mixed PCV schedules were received by 333 (79 %) and 315 (71 %) children, respectively. Pneumococcal (Spn) NP carriage was 76 % and 82 %, and non-typeable *Haemophilus influenzae* (NTHi) carriage was 68 % and 73 %, respectively. ED was obtained from 60 children (85 perforations) in the PCV7 group and from 47 children (59 perforations) in the PHiD-CV10 group. Data from bilateral perforations were combined. Spn was cultured from 25 % and 18 %, respectively, and NTHi was cultured from 61 % and 34 % respectively (*p* = 0.008).

**Conclusions:**

The observed reduction in the prevalence of suppurative OM in this population was not associated with reduced NP carriage of OM pathogens. The prevalence of NTHi-infected ED was lower in PHiD-CV10 vaccinated children compared to PCV7 vaccinated children. Changes in clinical severity may be explained by the action of PHiD-CV10 on NTHi infection in the middle ear. Randomised controlled trials are needed to answer this question.

**Electronic supplementary material:**

The online version of this article (doi:10.1186/s12887-015-0483-8) contains supplementary material, which is available to authorized users.

## Background

In October 2009 the PCV schedule for our region was changed from PCV7/PPV23 to PHiD-CV10 (Table 1). We recently reported a reduction in the prevalence of suppurative OM (either acute otitis media without perforation (AOMwoP), AOM with perforation (AOMwiP), or chronic suppurative OM (CSOM)) in PHiD-CV10-vaccinated children (39 %) compared to PCV7-vaccinated children (51 %) [1]. There was a concomitant increase in the prevalence of otitis media with effusion (OME) from 41 % in PCV7- to 51 % in PHiD-CV10-vaccinated children. Three key factors were found to be associated with the reduction in suppurative OM and increase in OME; i) PHiD-CV10 was protective compared to PCV7, ii) living in a household with more children less than 5 years of age per household was a risk factor, and iii) older age was protective of suppurative OM. Recent prescribing of antibiotics, child care attendance and other measured factors were not obviously associated with suppurative OM. Parents of the children in the survey were asked to consent to their child having a nasopharyngeal (NP) swab and a swab(s) of middle ear discharge (ED) if tympanic membrane perforation (TMP) was present (either AOMwiP or CSOM).

**Table 1:** Pneumococcal vaccines in the Childhood Vaccination Schedule for Northern Territory Indigenous children

Date commenced	Vaccine	Age (mo)
1^st^ July 2001	PCV7 & PPV23	2,4,6,18
1^st^ October 2009^a^	PHiD-CV10	2,4,6,18
1^st^ October 2011	PCV13	2,4,6,18

^a^No other Australian jurisdiction recommended that PHiD-CV10 replace PCV7

*Streptococcus pneumoniae* (pneumococcus, Spn) and non-typeable *Haemophilus influenzae* (NTHi) are major pathogens detected by culture [2, 3] and PCR [4] in ED of Australian Indigenous children with AOMwiP or CSOM. By culture, *H. influenzae, S. pneumoniae,* and *M. catarrhalis* were detected in 49 %, 33 % and 4 %, respectively of 55 ears diagnosed as AOMwiP whereas qPCR identified these pathogens in 89 %, 41 % and 18 %, respectively of ED swabs [4]. The reduction in prevalence of suppurative OM and concomitant increase in OME (i.e. relative improvement in OM severity) associated with the PHiD-CV10 schedule used in the NT could be mediated by differences in vaccines, either their component antigens or the level of immune protection in the middle ear [5]. PHiD-CV10 has an additional three pneumococcal serotypes (1, 5 and 7F) and potential NTHi protection due to conjugation of several serotypes with *H. influenzae* protein D [6]. It is also possible that the selective pressure of 10-valent vaccine may have further altered pneumococcal replacement and pneumococcal serotype distribution (although to date there is little evidence of this) [7]. Although the first pneumococcal protein D conjugate vaccine trial found efficacy of 11-valent pneumococcal protein D conjugate vaccine (11Pn-PD) against all-cause AOM, NTHi-AOM [6], and NTHi NP carriage [8], recently published PHiD-CV10 trials have shown no impact against NTHi NP carriage [7], or NTHi-AOM [9]. In the latter study, vaccine efficacy for all-cause AOM was 19 % [95 % CI 4.4 to 31.4] in intention-to-treat (ITT) analysis compared to 16 % [95 % CI-1.1 to 30.4] in according-to-protocol (ATP) analysis [9]. Our study compared the prevalence of all forms of OM and NP carriage of OM pathogens in young children receiving predominantly PCV7 or PHiD-CV10 according to NT childhood vaccination schedules. In this paper, we assess whether a change in severity of OM seen in the PHiD-CV10 era was accompanied by a change in the prevalence of OM pathogens in the NP or middle ear.

## Methods

### Study design, setting, community recruitment and ethical approval

This report includes cross-sectional data from 25 communities participating in at least one community-based, cross-sectional survey of OM and NP carriage between September 2008 and December 2012. The study was approved by the Human Research Ethics Committee of the NT Department of Health and the Menzies School of Health Research (EC00153), and the Western Australian Aboriginal Health Information and Ethics Committee (WAAHIEC). Each community council provided written approval of the study to the Ethics committee. Written informed consent was obtained from parents or carers for their child (regardless of ear health status or history) to have an ear examination, NP swab, swab of ED if present, and general child health check. Parents or carers were also asked to provide written permission to access the mother’s and the child’s medical records and to complete a lifestyle interview regarding information on likely risk factors for OM and NP carriage of OM pathogens. The Ethics committees approved this process and approved all participant recruitment processes, consent forms, participant information sheets and questionnaires.

### Participant recruitment and consent

In these remote communities where the birth cohort is between 5 and 45 infants per year, we aimed primarily to see all children under 36 months of age as well as older children (up to 6 years of age) if available. Individual families were approached with information about the study. Aboriginal children between 0 and 6 years of age, resident in participating communities, and whose parents or carers provided signed consent, were eligible for surveillance.

### Microbiology

NP swabs were collected, transported and stored as previously described [10] and in accordance with WHO recommendations for pneumococcal NP carriage studies [11]. Swab quality was also recorded as good (swab inserted to the NP and held for 5 s), fair (swab inserted partially and briefly), poor (swab of skin just below the nose) or very poor (of skin just below the nose and no discharge visible). Any swab with visible secretions was coded as good. Swabs of ED were collected after cleaning the external canal and collecting discharge from as close as possible to the TMP [10]. NP and ED swabs were cultured on selective and non-selective media and semi-quantitative colony counts recorded as previously described [10]. For ED swabs with swarming species that precluded selection of single NTHi colonies, a millipore filtration step was used [10]. At least 2 presumptive pneumococcal colonies and 2 presumptive NTHi colonies were selected from each specimen for confirmation. Colonies of minority colony morphology were chosen if present. Pneumococci were identified by colony morphology, optochin sensitivity and positive reaction with typing sera (Statens Serum Institut, Denmark); serotype was determined by Quellung reaction. NTHi were identified by colony morphology, dependence on X and V growth factors, and Phadebact agglutination. PCR discrimination of *H. haemolyticus* was not uniformly undertaken after confirmation that less than 0.2 % presumptive NTHi isolates from NP swabs in this population are misidentified [12]. Antimicrobial susceptibility was determined by the calibrated dichotomous susceptibility (CDS) disc diffusion method [13, 14]. Minimum inhibitory concentrations (MICs) were determined for macrolide and beta-lactam antibiotic resistance in *S. pneumoniae* isolates and azithromycin resistance in NTHi isolates using Etest strips (AB bioMérieux, Sweden). Beta-lactamase production by NTHi was determined using nitrocephin (Oxoid, Australia). Resistance was defined using European Committee on Antimicrobial Susceptibility Testing (EUCAST) breakpoints (http://www.eucast.org). Penicillin non-susceptibility of Spn was defined as MIC > 0.06 mg/L and azithromycin resistance as MIC > 0.5 mg/L. Azithromycin resistance in NTHi was defined as MIC > 4 mg/L; intermediate resistance as MIC > 0.12 mg/L and ≤ 4 mg/L, and susceptibility as MIC ≤ 0.12 mg/L.

### Clinical assessments

#### Ear examinations and general health assessments

All clinical assessments and diagnoses were made according to previously reported clinical outcomes of this surveillance [1]. In this report we describe the microbiology of ED from cases of AOMwiP or CSOM. We define AOMwiP as ED observed and TMP either recently healed or present for less than six weeks or covering less than 2 % of the pars tensa of the TM, and CSOM as ED observed and TMP present for longer than six weeks or covering at least 2 % of the pars tensa of the TM. We also refer to combination categories of any suppurative OM (any AOMwoP, AOMwiP or CSOM) and any TMP (any AOMwiP, dry perforation or CSOM). Where duration of discharge was not known, size of perforation was used to distinguish AOMwiP and CSOM. We asked the mother if she thought her child had ear pain that day or during the previous evening. These a priori diagnostic criteria have been applied in all our surveillance and clinical trials conducted in this population since 2001 [15].

#### Medical record review

The child’s medical records were reviewed to obtain dates of vaccinations, recent clinic presentations, antibiotics prescribed within the previous 5 weeks, sex, gestational age, date of birth, birth weight, and latest haemoglobin result.

### Risk factor questionnaires

The parent or guardian (usually the mother) was asked a standardised set of questions about common risk factors for OM, including the number of siblings, numbers of people and other children (less than 5 years of age) living in the child’s house, whether the mother or child’s siblings had ever had TMP (“runny ears”), her schooling and highest level of education, if she smoked, and whether the child was exposed to campfire smoke within the previous week, the number of days per week that the child attended child care, whether the child washed with soap the previous day, had ever used a pacifier, or was ever breastfed.

### Statistical analysis

Univariate analyses compared children who had received at least 2 doses of PCV7 with children who had received at least 2 doses of PHiD-CV10 as their primary course vaccines, with or without a subsequent single dose of an alternative PCV, or PPV23. Chi squared tests (and t-tests for several risk factors in Additional file 1: Table S1) were used. For this analysis we included only the first visit of children 0 to 36 months of age. Comparisons of children receiving non-mixed PCV schedules were also made. Confidence intervals (CI, 95 %) and risk differences (RD, 95 % CI) were calculated where appropriate. Stata version 12 was used for all data analyses [16].

Multivariate logistic regression analyses for pathogen-specific NP carriage and ED culture included all the risk factor and demographic variables for which significant *p* values (<0.05) were obtained for odds ratios in univariate analyses. Variables remained in the model if *p*<0.05. Where odds ratios are reported, they have come from logistic regression models, where adjustment for community was made using random effects. A global test was performed for categorical risk factors.

#### Missing data

If participants declined a clinical assessment or a swab, the data were coded as missing. If parents or carers refused or were unsure of their response to an interview question, we also coded this as missing. Missing data were then excluded from the denominator for summary statistics.

## Results

### Participant exclusions and PCV vaccination status of NP-swabbed children

As previously described [1], we enrolled 1,027 children and made 1,088 child visits in remote communities in the tropical Top End region of Australia; 895 children met inclusion criteria [1]. NP swabs were obtained from 421 of 444 (95 %) children in the PCV7 group and 443 of 451 (98 %) children in the PHiD-CV10 group. Non-mixed PCV schedules were received by 333 (79 %) and 315 (71 %) children, respectively. All 864 children included in this analysis had received two or more doses of appropriate vaccine and not more than one dose of any other PCV. Included and excluded schedules are detailed in Table 2.

**Table 2:** Number of doses of pneumococcal vaccine received by 421 NP-swabbed PCV7 children and 443 NP-swabbed PHiD-CV10 children

Doses of PCV7	Doses of PHiD-CV10					TOTAL
	0	1	2	3	4	
0	30^c^	18 ^c^	48	278	78	404
1	4 ^c^	2 ^c^	23	15	1	39
2	26	13	30 ^c^			39
3	305	72	1 ^c^			377^b^
4	4	1				5
+ PPV23^a^	132^a^					
+ PCV13^b^	2^b^	0	19^b^	69^b^	1^b^	91^b^
TOTAL	335	86	71	293	79	864

*PCV7* 7-valent pneumococcal conjugate vaccine

*PHiD*-*CV10* 10-valent pneumococcal *Haemophilus influenzae* protein D conjugate vaccine

*PCV13* 13-valent pneumococcal conjugate vaccine

Included mixed schedules:

^a^ 132 swabbed children also received one dose PPV23 (ceased in October 2010)

^b^ 91 swabbed children also received one dose PCV13:

2 of 305 PCV7 3-dose children

19 of 48 PHiD-CV10 2-dose children

69 of 278 PHiD-CV10 3-dose children

1 of 78 PHiD-CV10 4-dose child

Excluded schedules

^c^ 85 swabbed children were excluded, plus two additional children (not shown in Table 1) who received two doses of PHiD-CV10 and two doses of PCV13

### Participant characteristics, general health and antibiotics prescribed

Due to the high proportion of swabs collected, characteristics (e.g. age, gestational age, birth weight, gender, general health) of swabbed children in PCV7 and PHiD-CV10 groups (Table 3) were very similar to those previously described for these groups within the whole cohort [1]. As reported previously, the five largest communities contributed 59 % data to each group. Of note, the prevalence of any suppurative OM, antibiotics prescribed, runny nose and cough were the same in the subset of NP-swabbed children (Table 3) as was previously reported.

**Table 3:** Participant characteristics, general health and antibiotics prescribed, by vaccination group

	PCV7		PHiD-CV10		Absolute Difference in mean or % [95 % CI]	*P*
	n/N	Mean (SD) or %	n/N	Mean (SD) or %		
Age (mo)	421	20 (8)	443	18 (7)	2	0.0001
					[1 to 3]	
< 1 year	88	21 %	113	26 %		
1 to < 2 years	182	43 %	218	49 %		
2 to < 3 years	151	36 %	112	25 %		
Gestational age (weeks)	346	37.8 (2.8)	418	38.1 (2.2)	0.3	0.03
					[0 to 0.7]	
< 32 weeks	15	4 %	8/418	2 %		
Birth weight (kg)	358	2.97 (0.64)	423	3.06 (0.61)	0.08	0.03
					[0.00 to 0.17]	
< 3 kg	174	49 %	192	45 %		
Sex (female)	196/421	47 %	228/443	51 %	5 %	0.15
					[−2 to 12]	
GENERAL HEALTH & ANTIBIOTICS RECENTLY PRESCRIBED						
Runny Nose	160/413	39 %	195/437	45 %	6 %	0.08
					[−1 to 12]	
Any cough	80/416	19 %	169/439	38 %	19 %	<0.0001
					[13 to 25]	
Any suppurative OM	209/404	52 %	165/424	39 %	−13 %	0.0002
					[-20 to −6]	
Any antibiotics prescribed in previous 5 weeks	119/421	28 %	184/443	42 %	13 %	<0.0001
					[7 to 20]	
Beta-lactam	95/421	23 %	155/443	35 %	12 %	0.0001
					[6 to 18]	
Macrolide	13/421	3 %	18/443	4 %	1 %	0.44
					[−1 to 3]	
Topical	17/421	4 %	18/443	4 %	0 %	0.99
					[−3 to 3]	
Haemoglobin (<11 mg/dL)	115/385	30 %	137/398	34 %	5 %	0.17
					[−2 to 11]	

### NP carriage

NP swabs from four of 864 children were not processed. No significant differences between the PCV7 and PHiD-CV10 groups were found for carriage prevalence of NTHi (68 % and 73 %, respectively), Spn (76 % and 82 %) or *Moraxella catarrhalis* (Mcat) (48 % and 43 %) or NTHi and Spn combined carriage (64 % and 68 %). *Staphylococcus aureus* (Sa) NP carriage was significantly lower in the PHiD-CV10 group (32 % and 21 %), and Mcat was lower when non-mixed schedules were compared (Table 4). Most children (86 % and 91 %, respectively) carried one or other of these pathogens. A comparison of NP carriage among children who received non-mixed PCV schedules gave similar results, although the difference between groups in Mcat was more pronounced (Table 4).

**Table 4:** Prevalence of NP carriage of OM pathogens in NP-swabbed children, by vaccination group

	PCV7		PHiD-CV10		Absolute Risk Difference	*P* value
					[95 % CI]	
	n/N	%	n/N	%		
NP carriage						
NTHi	284/420	68 %	322/440	73 %	6 %	0.07
					[−0.5 to 12]	
Spn	321/420	76 %	359/440	82 %	5 %	0.06
					[−0.2 to 11]	
Mcat	202/420	48 %	189/438	43 %	−5 %	0.15
					[−12 to 2]	
Sa	136/420	32 %	94/440	21 %	−11 %	0.0003
					[−17 to −5]	
Combination NP carriage categories						
Spn and NTHi	267/420	64 %	297/440	68 %	4 %	0.23
					[−2 to 10]	
Spn or NTHi	361/420	86 %	400/440	91 %	5 %	0.02
					[0.6 to 9]	
PCV7 types	38/319	12 %	31/372	8 %	−4 %	0.12
					[−8 to1]	
PCV13 types	92/319	29 %	77/372	21 %	−8 %	0.01
					[−15 to −2]	
Serotypes^b^	16F^cd^(12 %) 19A^cd^(10 %) 11A^cde^ 23B^cde^ 6C^c^ = 19F^de^ 23F^cde^ = 6A^a^ = 10A^d^ 33F		16F^d^(14 %) 19A^de^(8 %) 11A^de^ 10A^d^ 6C^c^ 15A^de^ 23F^cde^ 21^c^ 23B^d^ 35F			
	Non-mixed PCV schedules					
NP carriage	333^a^		314			
NTHi	225/332	68 %	230/314	73 %	5 %	0.13
					[−2 to 12]	
Spn	253/332	76 %	259/314	82 %	6 %	0.05
					[0 to 12]	
Mcat	172/332	52 %	127/313	41 %	−11 %	0.004
					[−19 to −4]	
Sa	105/332	32 %	70/314	22 %	−9 %	0.008
					[−16 to −2]	

^a^125 children in the PCV7-only group had also received PPV23

^b^Strains within serotypes that are:

^c^ Azithromycin non-susceptible (MIC > 0.5 mg/L)

^d^ Penicillin non-susceptible (MIC > 0.06 mg/L)

^e^ Non-susceptible to both

### Pneumococcal serotypes in the NP

**Fig. 1:**
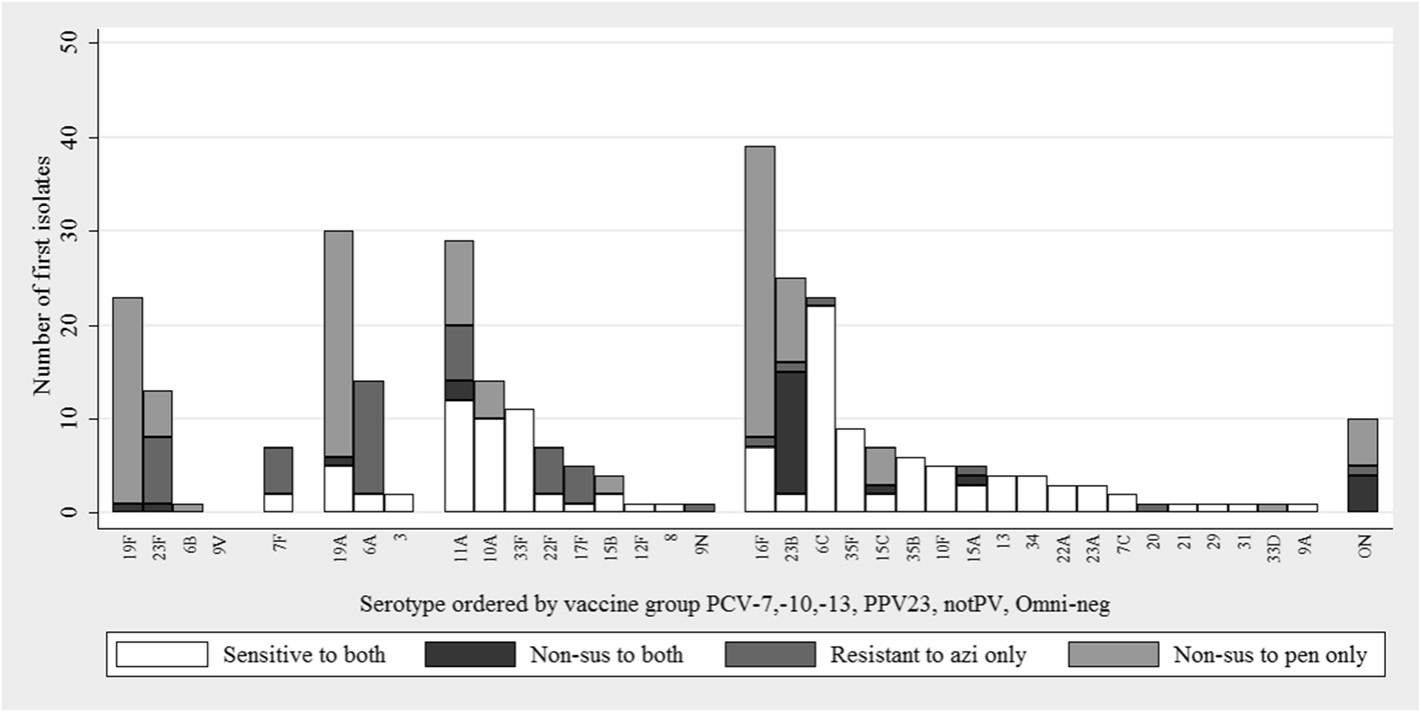
Pneumococcal serotypes and antimicrobial susceptibility of isolates colonising the nasopharynx of the PCV7 group. The first isolate selected per NP swab is included. Serotype is ordered by pneumococcal vaccine group: PCV7, PHiD-CV10, PCV13, 23PPV and non-vaccine types (notPV and Omniserum-negative). Penicillin non-susceptibility was defined as MIC > 0.06 mg/L. Azithromycin resistance was defined as MIC > 0.5 mg/L

**Fig. 2:**
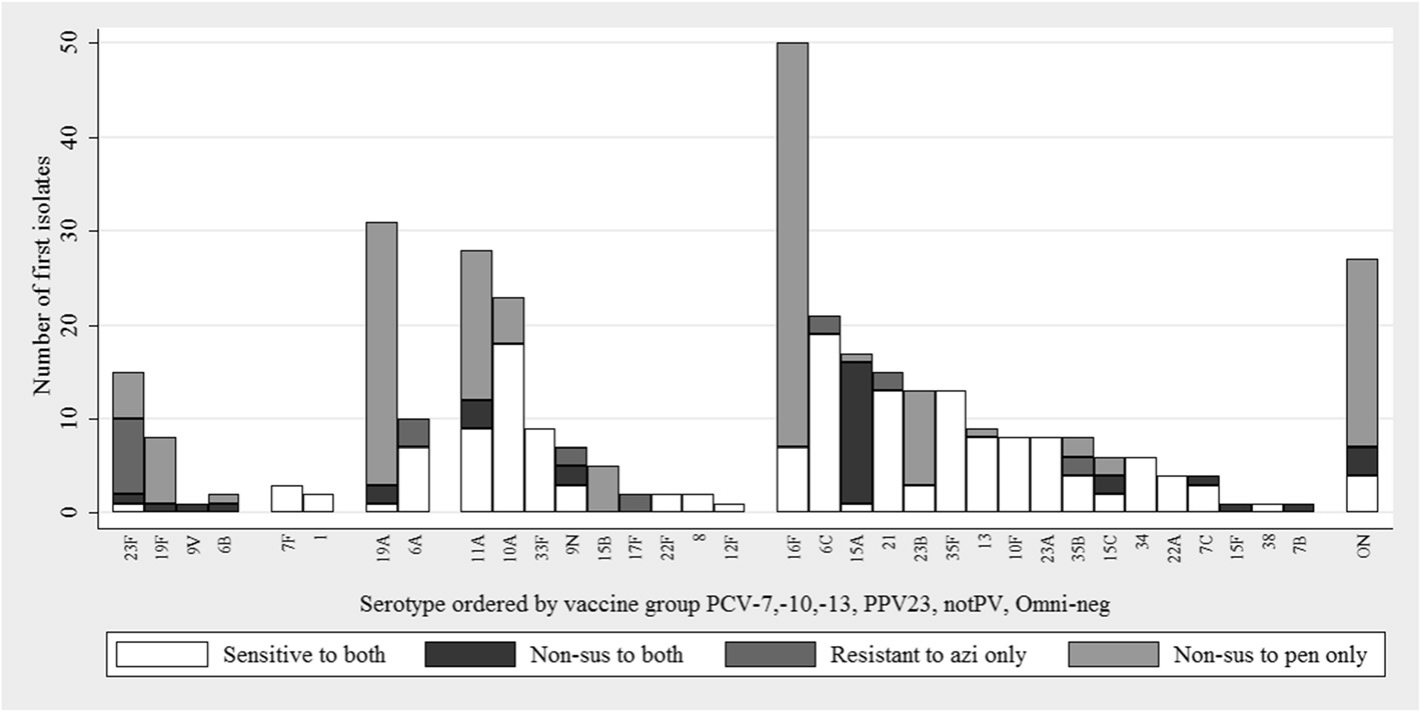
Pneumococcal serotypes and antimicrobial susceptibility of isolates colonising the nasopharynx of the PHiD-CV10 group. The first isolate selected per NP swab is included. Serotype is ordered by pneumococcal vaccine group: PCV7, PHiD-CV10, PCV13, 23PPV and non-vaccine types (notPV and Omniserum-negative). Penicillin non-susceptibility was defined as MIC > 0.06 mg/L. Azithromycin resistance was defined as MIC > 0.5 mg/L

The most frequent colonisers in the PCV7:PHiD-CV10 groups respectively, were 16F (12 % :14 %) 19A (10 %:8 %), and 11A (9 %:8 %). Serotypes 23B, 19F and 6C were the next most frequent colonisers in the PCV7 group; 10A, 6C and 15A in the PHiD-CV10 group. PCV13 serotypes colonised fewer children in the PHiD-CV10 group (21 %) than the PCV7 group (29 %) (Table 4 and Figs. 1 and 2). In the PCV7 group 39 % (128/326) Spn isolates were susceptible to both penicillin and azithromycin, 37 % were penicillin non-susceptible, 14 % were azithromycin non-susceptible and a further 8 % were non-susceptible to both. In the PHiD-CV10 group, 44 % (163/373) Spn isolates were susceptible to both penicillin and azithromycin, 39 % were penicillin non-susceptible, 6 % were azithromycin non-susceptible, and a further 9 % were non-susceptible to both.

### NP swab quality

NP swab quality was recorded for 393 of 421 (93 %) and 361 of 443 (81 %) NP swabs collected from PCV7 and PHiD-CV10 children, respectively. Most swabs (92 %) were good or fair quality, 2 were very poor quality; 23 were swabs of secretions collected by nose blowing into a tissue. Recovery of NTHi, Spn or Sa was not significantly different for different quality swabs or swabs with missing data on quality.

### Recovery of OM pathogens from ED

We collected 144 ED swabs from 60 children (85 ears) in the PCV7 group and 47 children (59 ears) in the PHiD-CV10 group (Table 5). Despite use of selective media and filtration methods [10], overgrowth by swarming species on agar plates (hereafter referred to as masking) affected recovery of NTHi from 12 EDs in the PCV7 group and 4 in the PHiD-CV10 group, Spn from 2 and 3, and Mcat from 24 and 4, respectively. Of the ED swabs where recovery of NTHi was not affected by masking, NTHi was cultured from a greater proportion of EDs in the PCV7 group (53 %) compared to the PHiD-CV10 group (35 %). Similarly, Spn was cultured from 24 % and 14 %, Mcat from 2 % and 7 %, and Sa from 54 % and 43 %, respectively. When data from children with bilateral discharge were combined, results were similar, although the difference in NTHi was more pronounced (RD −27 % [95 % CI −46 to −8] *p* = 0.008) (Table 5). We found no significant differences in recovery of any OM pathogen from EDs from AOMwiP compared to CSOM (data not shown).

**Table 5:** Prevalence of OM pathogens in 144 ear discharge (ED) swabs from 107 children with AOMwiP or CSOM, by vaccination groups

	PCV7		PHiD-CV10		Absolute Risk Difference [95 % CI]	*P* value
	N, Mean (SD)	%	N, Mean (SD)	%		
ED swabs	85		59			
Children with ED	60^a^		47			
Mean age (mo)	20 (7.6)		18 (6.9)			
Bilateral ED swabs	19		13			
CSOM	11		8			
AOMwiP	6		3			
AOMwiP and CSOM	2		2			
All ED swabs	85		59			
> =3-dose schedule	77/85	91 %	53/59	90 %		
One dose PCV13	0		9/59	15 %		
NTHi	39/73^m-^	53 %	19/55	35 %	−19 %	0.03
					[−36 to −2]	
Spn	20/83	24 %	8/56	14 %	−10 %	0.16
					[−23 to 3]	
NTHi and Spn	12/69	17 %	5/55	9 %	−8 %	0.18
					[−20 to 3]	
Mcat	1/61	2 %	4/55	7 %	6 %	0.14
					[−2 to 13]	
Sa	44/82	54 %	24/56	43 %	−11 %	0.21
					[−28 to 6]	
Spn serotypes^b^	10A(*n* = 4) 7F^c^(*n* = 2) 11C^d^(*n* = 2) 10F 12F 16F^d^ 17F^c^ 19A^d^ 19F^d^ 23F^c^ 6A 6C		11A^d^(*n* = 2) 15A^c^ 16F^d^ 19F^d^ 21 22A 35F			
Children with ED (Bilateral data combined)	60		47			
> =3 dose schedule	54/60	90 %	42/47	89 %		
One dose PCV13	0		9/47	19 %		
NTHi	33/54^m-^	61 %	15/44	34 %	−27 %	0.008
					[−46 to −8]	
Spn	15/60	25 %	8/45	18 %	−7 %	0.38
					[−23 to 8]	
NTHi and Spn	11/60	18 %	5/45	11 %	−7 %	0.31
					[−21 to 6]	
Mcat	1/46	2 %	3/44	7 %	5 %	0.27
					[−4 to 13]	
Sa	35/59	59 %	22/45	49 %	−10 %	0.29
					[−30 to 9]	

^a^ 53 children in the PCV7 group had also received PPV23

^b^ Strains within this serotype are:

^c^ Azithromycin non-susceptible (MIC > 0.5 mg/L)

^d^ Penicillin non-susceptible (MIC > 0.06 mg/L)

^e^ Non-susceptible to both

^f-^ ED culture not affected by masking

### Pneumococcal serotypes in ED

Isolates from three of four bilateral Spn-positive EDs were the same serotype, and were included once in analysis. We serotyped 27 first isolates, 20 second, 3 third and 3 fourth isolates from the 30 Spn-positive ED specimens. Only one of 26 second, third or fourth isolates, a serotype 19A, was different to the first isolate, an 11A. Of the 27 first isolates serotyped, 17 were from the PCV7 group and 10 from the PHiD-CV10 group. Overall, 17 serotypes were detected. The hierarchy of 12 serotypes in the PCV7 group was 10A (*n* = 4), 7F (*n* = 2), 11C (*n* = 2) and one each of 10F, 12F, 16F, 17F, 19A, 19F, 23F, 6A and 6C. The hierarchy of seven serotypes in the PHiD-CV10 groups was 11A(*n* = 2) and one each of 15A, 16F, 19F, 21, 22A and 35F. Five of the seven serotypes in the PHiD-CV10 group had not been detected in the PCV7 children and 10 of the 12 serotypes in the PCV7 group were not detected in the PHiD-CV10 group; only 16F and 19F were common.

### 23-valent pneumococcal polysaccharide vaccine (PPV23)

PPV23 was received by 132 NP-swabbed children in the PCV7 group. In a post-hoc analysis, we previously reported that the PCV7-PPV23 group had a higher proportion of TMP than PCV7-only children. Compared to 38 age-eligible children who received PCV7-only, NP carriage of Spn in the PCV7-PPV23 group was not significantly different (77 % in the PCV7-PPV23 group and 76 % in the PCV7-only group). Similarly for ED swabs, 11 of 40 (28 %) ED swabs from children with PPV23 vaccination cultured Spn compared to 1 of 5 (20 %) not PPV23 vaccinated.

### Risk factors

#### Questionnaire response

Of the 421 NP-swabbed children in the PCV7 group, 392 (93 %) parents or carers consented to a structured risk factor questionnaire conducted by interview, whereas only 289 (65 %) parents or carers of the 443 NP-swabbed children in the PHiD-CV10 group consented to the interview. Despite these differences in response rates, NP carriage among responders was similar to NP carriage in the whole NP-swabbed cohort. Furthermore, within the PHiD-CV10 group, NP carriage was similar for the consented and non-consented children (NTHi: 71 % versus 76 %, respectively; Spn: 82 % versus 80 %, respectively).

#### Prevalence of risk factors

Similar risk factor prevalence rates were found for the NP-swabbed children (Additional file 1: Table S1) as previously reported [1]. Briefly, the mean number of children less than 5 years of age per household was lower in the PHiD-CV10 group. Other risk factors were comparable between PCV7 and PHiD-CV10 groups.

### Risk factors associated with NP carriage of (i) NTHi and (ii) Spn

In univariate analyses, vaccine group was not associated with lower NP carriage of NTHi or Spn (Table 6). The number of children less than 5 years of age in the household was associated with increased risk of NTHi carriage, maternal schooling was protective, and low haemoglobin level (<11 mg/l) was a risk factor. In the multivariate analyses (data not shown) with these variables, maternal schooling and low haemoglobin were significantly associated with NP carriage of NTHi. Adjustment for age made no substantial difference to these findings. Pacifier use and recent antibiotic prescribing were protective of Spn carriage, and breast feeding was a risk factor in the univariate analyses. In the multivariate analyses, breast feeding and pacifier use remained significant. After adjustment for age, both dropped out of the model (the effect of PHiD-CV10 remained similar to that reported in Table 6).

**Table 6:** Univariate odds ratios for risk factors for NP carriage of NTHi or Spn in 860 children, adjusted for community

	NTHi				Spn			
Risk Factor (n)		OR	95 % CI	*P*		OR	95 % CI	*P*
Vaccine group (*n* = 860)								
PCV7	68 %				76 %			
PHiD-CV10	73 %	1.3	0.96 to 1.78	0.09	82 %	1.4	1.00 to 2.06	0.05
Age group (*n* = 860)								
< 1 year	71 %			0.98	77 %			0.48
1 to < 2 years	71 %	0.98	0.67 to 1.43		79 %	1.09	0.71 to 1.66	
2 to < 3 years	70 %	0.94	0.62 to 1.43		81 %	1.30	0.81 to 2.09	
Gender (*n* = 860)								
Male	70 %				78 %			
Female	71 %	1.00	0.74 to 1.34	0.98	80 %	1.11	0.79 to 1.57	0.53
Gestational age (*n* = 760)								
> = 32 weeks	71 %				81 %			
< 32 weeks	65 %	0.72	0.30 to 1.75	0.47	65 %	0.40	0.16 to 0.98	0.05
Antibiotics prescribed in previous 5 weeks (*n* = 860)								
None	69 %				82 %			
Any	74 %	1.30	0.95 to 1.79	0.11	73 %	0.58	0.41 to 0.82	0.002
Crowding (number of additional children less than 5 years of age in household) (*n* = 670)								
0	67 %			0.04	75 %			0.25
1	69 %	1.12	0.76 to 1.66		79 %	1.29	0.83 to 2.00	
2	61 %	0.74	0.44 to 1.26		79 %	1.19	0.64 to 2.22	
3+	79 %	1.70	0.97 to 2.36		85 %	1.62	0.83 to 2.23	
Child care attendance (*n* = 673)								
< 3 days per week	69 %				78 %			
3 or more days per week	72 %	1.18	0.70 to 1.99	0.53	82 %	1.34	0.71 to 2.52	0.37
Sibling history of OM (“runny ears”) (*n* = 618)								
No	68 %				80 %			
Yes	70 %	1.11	0.75 to 1.63	0.61	77 %	0.91	0.59 to 1.41	0.68
Child near campfire last week (*n* = 659)								
No	70 %				77 %			
Yes	68 %	0.90	0.63 to 1.28	0.56	81 %	1.36	0.89 to 2.09	0.16
Haemoglobin (*n* = 780)								
> =11 mg/dL	68 %				79 %			
<11 mg/dL	76 %	1.45	1.02 to 2.07	0.04	80 %	1.04	0.70 to 1.53	0.86
Maternal smoking (*n* = 673)								
No	68 %				79 %			
Yes	69 %	1.05	0.75 to 1.48	0.76	78 %	0.97	0.65 to 1.44	0.88
Maternal schooling (*n* = 528)								
Year 10 or less	73 %				78 %			
Year 11 or 12	63 %	0.65	0.44 to 0.95	0.03	77 %	0.94	0.61 to 1.47	0.80
Maternal age at birth of this child (*n* = 649)								
> = 21 yrs	69 %				79 %			
< 21 yrs	72 %	1.17	0.80 to 1.71	0.42	80 %	1.09	0.70 to 1.69	0.69
Breastfed (*n* = 678)								
Never	60 %				65 %			
Some	69 %	1.53	0.78 to 2.99	0.21	79 %	2.34	1.14 to 4.80	0.02
Pacifier (*n* = 671)								
Never	70 %				80 %			
Some	65 %	0.81	0.53 to 1.25	0.34	70 %	0.52	0.33 to 0.84	0.007
PPV23 (*n* = 163)^a^								
no	66 %				76 %			
yes	74 %	1.5	0.69 to 3.3	0.30	77 %	1.1	0.45 to 2.7	0.83

^a^163 children > = 18 months of age

### Risk factors and ED microbiology

No risk factors (other than PHiD-CV10 for NTHi) were associated with the reduced culture of NTHi or Spn from ED.

## Discussion

We recently reported a reduction in the prevalence of suppurative OM and a concomitant increase in OME prevalence in PHiD-CV10-vaccinated children compared to PCV7-vaccinated children [1]. Our parallel NP carriage surveillance and opportunistic collection of ED from children with either AOMwiP or CSOM provide data to help interpret clinical findings.

We report here that the NP carriage prevalence of NTHi and Spn was fairly similar in the PHiD-CV10 group compared to the PCV7 group. Most children had received three or more doses of one PCV, and a comparison of non-mixed schedules was consistent with the lack of difference between PCV groups. This is in contrast to NP carriage of Mcat and Sa, two minor OM pathogens, which were lower in the PHiD-CV10 group compared to the PCV7 group. In the multivariate logistic regression analyses, maternal schooling (year 11 or 12 completion compared to year 10 or less) was associated with less NTHi NP carriage, and low haemoglobin was a risk factor. Anaemia is very common among Aboriginal children in remote communities [17] and is an important risk factor for acute lower respiratory tract infection in developing countries [18].

Our data on the microbiology of ED collected from children with either AOMwiP or CSOM are particularly interesting as they describe a reduction in NTHi culture from the ED of PHiD-CV10-vaccinated children compared to PCV7-vaccinated children. Furthermore, there was no vaccine group difference in Spn culture from EDs, or other OM pathogens. This suggests that there may be a compartmental effect of PHiD-CV10-induced immune response in the ear compared to the NP.

Whilst we understand the NP to be the source of pathogens that cause middle ear infections, we suspect that vaccine-induced immune responses could deliver protection in the middle ear, where numbers of organisms are likely to be lower, without eliminating NP carriage. This hypothesis is plausible (not all NP colonised children have OM), and should be confirmed in randomised controlled vaccine trials with OM outcomes and concomitant studies of NP and, if possible, MEF microbiology. Indeed the 11Pn-PD trial (POET study) showed a significant 35 % reduction in NTHi-positive MEF from children vaccinated with 11Pn-PD compared to controls [6]. A significant reduction in NP carriage was also recorded, but only three months following the booster (4th) dose [8]. The data from PHiD-CV10 have been less convincing with no significant impact on NP carriage of NTHi being reported from an observational study [7] or a randomised controlled trial (RCT) [19]. The latter RCT included analyses of colonisation density which was also not different between PHiD-CV10 and PCV7 groups. In Kilifi, analysis of NP carriage prior to and following PHiD-CV10 introduction to the national vaccination schedule found NP carriage of NTHi in children under 5 years of age was 54 % before PHiD-CV10 introduction and 40 % after introduction (adjusted prevalence ratio 0.62) [20]. Almost no study, including the RCT above, has been able to analyse middle ear fluid from PCV7- compared to PHiD-CV10-vaccinated children, so more data are needed to support the notion that OM can be prevented whilst children remain colonised. Data from animal studies show a site-specific effect of protein D-dependent adherence [21] which the authors attributed to the compartment-specific host cell type; epithelial tissue in the nasopharynx being predominantly ciliated columnar, whereas cells in the middle ear are primarily non-ciliated and squamous. It was also suggested that load of colonising NTHi may be reduced by anti-protein D antibodies, limiting disease development [21]. Failure of PHiD-CV10 to eradicate or protect from NP carriage of NTHi precludes benefits of herd effects for the non-vaccinated population. At the same time, this could be advantageous in terms of maintaining the integrity of the NP flora, particularly if sterile sites can be protected.

There remains the possibility that PHiD-CV10 has more subtly influenced the NP microbiota. Not all NTHi strains have the *hpd* gene for protein D expression, the NTHi immunogenic target of PHiD-CV10 [22]. Thus vaccine selective pressures may apply with consequent shifts in the NTHi population. We found that NP carriage of Sa was significantly lower in PHiD-CV10-vaccinated children, which was not replicated in the middle ear cultures. The NP carriage studies by van den Bergh et al. and Hammitt et al. found no significant impact of PHiD-CV10 on NP carriage of Sa [19, 20]. Further work needs to be done to establish impacts of vaccination on the human microbiome.

There was no substantial difference in the serotypes colonising the NP of PCV7 compared to PHiD-CV10-vaccinated children. There was a high level of serotype diversity with less than 30 % being PCV13 serotypes. Serotypes 16 F and 19A accounted for 22 % isolates in each vaccine group. Serotype 16F has been predominant in NP carriage of adults and children in this population over decades [23, 24]. In the ED, only serotypes 16F and 23F were common to both vaccine groups. Serotype diversity was high and numbers too low to make meaningful comparisons, or to suggest that a clinical difference may be associated with the shift in serotypes. Interestingly, in a large study in Israel during PCV7 and PCV13 eras, serotype 16F has become the second most common non-PCV serotype isolated by tympanocentesis from MEF of children with AOM [25]. Serotype 3 rarely colonises the NP, but is an important cause of AOM. Around 30 % pneumococci recovered from ED of German PCV7-vaccinated children with spontaneous otorrhoea were serotype 3 [26]. Serotype 3 was not included in the PHiD-CV10 formulation following negative efficacy in the pre-licensure 11Pn-PD vaccine [6]. Although PCV7 does not include serotype 3, reductions in serotype 3 AOM incidence in Israel were seen during the PCV7 era, but interestingly no further reductions were seen in the PCV13 era [25]. We did not find serotype 3 in the EDs of PCV7- or PHiD-CV10-vaccinated children in this study.

Previously our group has enhanced detection of NTHi, Spn and Mcat in the NP [27] and ED [4] by applying PCR. Culture results for ED swabs from 55 children with AOMwiP were 49 % NTHi, 33 % Spn and 4 % Mcat whereas qPCR identified 89 %, 41 % and 18 % respectively, in ED swabs [4]. For children with OME there is generally a low rate of culture positivity although NTHi (8 %) and Mcat (5 %) are dominant [28]. We are not able to confirm the proportion of OME cases in Aboriginal children that might be culture positive, but as we detected an increase in OME in PHiD-CV10-vaccinated children compared to PCV7-vaccinated children, it is plausible that NTHi persists in the middle ears of these children, but in a metabolically altered state (for example, in biofilm). Data from non-Indigenous Australian children with recurrent AOM also show a dominance of NTHi in middle ear effusions by culture (13 %) and PCR (47 %), compared to Spn (6 % and 6 %, respectively) and Mcat (6 % and 13 %, respectively) [29]. Similar dominance of NTHi in the MEF of children undergoing tympanostomy tube insertion was reported from New Zealand [30] and in children with recurrent AOM in Spain [31].

## Conclusions

This comparison of NP carriage and ED microbiology in children vaccinated with PCV7 or PHiD-CV10 (2 or more doses, or non-mixed schedules) found no difference in the prevalence of Spn or NTHi NP carriage. For children with tympanic membrane perforation (AOMwiP or CSOM), fewer PHiD-CV10-vaccinated children had NTHi culture-positive ED than PCV7-vaccinated children. It is plausible that all PHiD-CV10-vaccinated children, including those with AOMwoP, had less NTHi in the middle ear. This would be consistent with our finding that fewer PHiD-CV10-vaccinated children had suppurative OM (AOMwoP, AOMwiP or CSOM) than PCV7-vaccinated children [1]. Whether the reduction in NTHi in ear discharge is due to protection elicited by the NTHi protein D of the PHiD-CV10 vaccine or other factors is not known. This question is best answered through the conduct of well-designed RCTs that include microbiological outcome measures.

Synflorix® is a trademark of the GlaxoSmithKline group of companies.

Prevenar® and Prevenar 13® are trademarks of Pfizer Inc.

## Additional file

Additional file 1:
**Table S1.** Prevalence of risk factors, OM and NP carriage among a subgroup of NP-swabbed children whose parents consented to a lifestyle questionnaire, by vaccination group. (DOC 51 kb)

## Abbreviations

AOMwiP: Acute otitis media with perforation
AOMwoP: Acute otitis media without perforation
ATP: According to Protocol
CDS: Calibrated Dichotomous Susceptibility
CI: Confidence Interval
CSOM: chronic suppurative otitis media
DP: Dry perforation
ED: Ear discharge
*Hpd*: *Haemophilus influenzae* protein D gene
IPD: Invasive pneumococcal disease
ITT: Intention to Treat
Mcat: *Moraxella catarrhalis*
MIC: Minimum inhibitory concentration
Mo: Month
NP: Nasopharynx
NT: Northern Territory
NTHi: Non-typeable *Haemophilus influenzae*
OM: Otitis media
OME: Otitis media with effusion
PCR: Polymerase Chain Reaction
PCV7: 7-valent pneumococcal conjugate vaccine
PCV13: 13-valent pneumococcal conjugate vaccine
PHiD-CV10: 10-valent pneumococcal *Haemophilus influenzae* protein D conjugate vaccine
PPV23: 23-valent pneumococcal polysaccharide vaccine
RD: Risk Difference
Sa: *Staphylococcus aureus*
SD: Standard deviation
Spn: *Streptococcus pneumoniae*
TMP: Tympanic membrane perforation
