# Supplementary material for: Reduced middle ear infection with non-typeable Haemophilus influenzae, but not Streptococcus pneumoniae, after transition to 10-valent pneumococcal non-typeable H. influenzae protein D conjugate vaccine
Source: BMC Pediatr. 2015 Oct 19;15:162. doi: 10.1186/s12887-015-0483-8 (PMC4615539; doi:10.1186/s12887-015-0483-8)
Supplement: Additional file 1: — Table S1. Prevalence of risk factors, OM and NP carriage among a subgroup of NP-swabbed children whose parents consented to a lifestyle questionnaire, by vaccination group. (DOC 51 kb) [file 12887_2015_483_MOESM1_ESM.doc]

**Additional file 1: Table S1. Prevalence of risk factors, OM and NP carriage among a subgroup of NP-swabbed children whose parents consented to a lifestyle questionnaire, by vaccination group**

|  | **PCV7** | | **PHiD-CV10** | | **Absolute Difference in mean or %**  **[95% CI]** | **p** |
| --- | --- | --- | --- | --- | --- | --- |
|  | **N** | **Mean (SD) or %** | **N** | **Mean (SD) or %** |  |
| RISK FACTOR QUESTIONNAIRE RESPONSE | | | | | | |
| Consent not documented | 4/421 | 1% | 10/443 | 2% | 1%  [-0.3 to 3] | 0.13 |
| Number of parents consented | 392/417 | 94% | 289/433 | 67% | -27%  [-32 to -22] | **<0.0001** |
| Mother interviewed | 381/392 | 97% | 283/289 | 98% | 1%  [-2 to 3] | 0.55 |
| CHARACTERISTICS OF CHILDREN in CONSENTED SUBGROUPS | | | | | | |
| Age (months) | 392 | 20 (8) | 289 | 18(7) | 2.0  [0.8 to 3.2] | **0.0005** |
| Sex (female) | 186 | 47% | 148 | 51% | 4%  [-4 to 11] | 0.33 |
| Any Suppurative OM | 202/377 | 54% | 100/278 | 36% | -18%  [-25 to -10] | **<0.0001** |
| Any NTHi carriage | 263/391 | 67% | 205/287 | 71% | 4%  [-3 to 11] | 0.25 |
| Any Spn carriage | 296/391 | 76% | 235/287 | 82% | 6%  [0 to 12] | 0.05 |
| RISK FACTORS for NP CARRIAGE in CHILDREN in CONSENTED SUBGROUPS | | | | | | |
| Crowding  (number of persons in household) | 382 | 8.7 (4.6) | 283 | 8.2 (3.9) | -0.5  [-1.1 to 0.2] | 0.09 |
| Crowding  (number of additional children less than 5 years of age in household) | 388 | 2.4 (1.6) | 284 | 2.1 (1.2) | -0.3  [-0.5 to -0.1] | **0.01** |
| Crowding Index (% households with > 2 additional children < 5 years of age) | 125/388 | 32% | 71/284 | 25% | -7%  [-14 to -0.2] | **0.04** |
| Child Care  (days/week) | 388 | 0.64 (1.6) | 288 | 0.78 (1.7) | 0.15  [-0.11 to 0.40] | 0.13 |
| Child Care  (any attendance >= 3 days per week) | 44/388 | 11% | 41/288 | 14% | 2.9%  [-2 to 8] | 0.26 |
| Washed with soap yesterday | 309/343 | 90% | 251/286 | 88% | -2%  [-7 to 3] | 0.35 |
| Sibling history of OM (“runny ears”) | 103/360 | 29% | 76/246 | 31% | 2%  [-5 to 10] | 0.20 |
| Child near campfire last week | 131/382 | 34% | 87/280 | 31% | -3%  [-10 to 4] | 0.38 |
| Maternal smoking | 221/390 | 57% | 172/286 | 60% | 3%  [-4 to 11] | 0.37 |
| Maternal age at birth of this child | 284 | 25 (5.4) | 239 | 26 (6.4) | 1  [0.1 to 2] | **0.01** |
| Maternal age less than 21 years | 94/310 | 30% | 98/341 | 29% | -2%  [-9 to 5] | 0.66 |
| Maternal education (certificate) | 99/363 | 27% | 57/240 | 24% | -4%  [-11 to 4] | 0.33 |
| Maternal education (year 11 or 12) | 168/317 | 53% | 127/214 | 59% | 6%  [-2 to 15] | 0.15 |
| Never breast fed | 27/409 | 7% | 14/292 | 5% | -2%  [-6 to 1] | 0.24 |
| Pacifier (ever) | 75/385 | 19% | 49/289 | 17% | -3%  [-8 to 3] | 0.40 |
